# Defining a minimal clinically important difference for endometriosis-associated pelvic pain measured on a visual analog scale: analyses of two placebo-controlled, randomized trials

**DOI:** 10.1186/1477-7525-8-138

**Published:** 2010-11-24

**Authors:** Christoph Gerlinger, Ulrike Schumacher, Thomas Faustmann, Antje Colligs, Heinz Schmitz, Christian Seitz

**Affiliations:** 1 Global Clinical Statistics, Bayer Schering Pharma AG, 13342 Berlin, Germany; 2 Biometry, Jenapharm GmbH & Co. KG, and Zentrum für Klinische Studien, Universitätsklinikum Jena, 07743 Jena, Germany; 3 Global Medical Affairs Women's Healthcare, Bayer Schering Pharma AG, 13342 Berlin, Germany; 4 Global Market Access, Bayer Schering Pharma AG, 13342 Berlin, Germany; 5 Global Clinical Development Women's Healthcare, Bayer Schering Pharma AG, 13342 Berlin, Germany

## Abstract

**Background:**

When comparing active treatments, a non-inferiority (or one-sided equivalence) study design is often used. This design requires the definition of a non-inferiority margin, the threshold value of clinical relevance. In recent studies, a non-inferiority margin of 15 mm has been used for the change in endometriosis-associated pelvic pain (EAPP) on a visual analog scale (VAS). However, this value was derived from other chronic painful conditions and its validation in EAPP was lacking.

**Methods:**

Data were analyzed from two placebo-controlled studies of active treatments in endometriosis, including 281 patients with laparoscopically-confirmed endometriosis and moderate-to-severe EAPP. Patients recorded EAPP on a VAS at baseline and the end of treatment. Patients also assessed their satisfaction with treatment on a modified Clinical Global Impression scale. Changes in VAS score were compared with patients' self-assessments to derive an empirically validated non-inferiority margin. This anchor-based value was compared to a non-inferiority margin derived using the conventional half standard deviation rule for minimal clinically important difference (MCID) in patient-reported outcomes.

**Results:**

Anchor-based and distribution-based MCIDs were-7.8 mm and-8.6 mm, respectively.

**Conclusions:**

An empirically validated non-inferiority margin of 10 mm for EAPP measured on a VAS is appropriate to compare treatments in endometriosis.

## Introduction

Endometriosis is a common condition in women of reproductive age that is characterized by the presence of functional endometrium-like tissue outside the uterus (e.g., the ovaries and other pelvic structures). Changes in the number and size of such endometriotic lesions were often used to assess the efficacy of treatment options for endometriosis [1-4]. However, there is no direct correlation between the extent of these lesions and the severity of the symptoms experienced by the patient [5-7]. Potential explanations for this lack of correlation are that the level of pain induced by endometriosis might be determined by the depth of tissue intrusion of a specific lesion, or that there may be a direct interaction of endometriotic lesions and nerve fibers [8,9]. Neither of these potential explanations can be assessed by visual inspection during surgery and are therefore not reflected in the respective scoring systems for endometriosis severity [10,11].

Typical symptoms of endometriosis include dysmenorrhea, dyspareunia, and chronic pelvic pain [12-14]. Pain is commonly considered the most relevant symptom and the primary reason for treatment [14,15]. Different tools for assessing pain in endometriosis, such as the visual analog scale (VAS) or numerical rating scales, have been used in the past. Recommendations on how to assess endometriosis-related symptoms in a way that allows for comparison of results between clinical trials have recently been published [16]. However, publications on the validity of the pain and quality-of-life scales for use in endometriosis are still scarce and information on the psychometric properties of such tools in the indication of endometriosis are generally lacking [17,18].

When comparing the efficacy of different active treatments in endometriosis-associated pelvic pain (EAPP), a non-inferiority-also called a one-sided equivalence-study design is often used. This is because a new treatment modality might provide a benefit for the patient (e.g., better tolerability and/or safety) even if it is not superior to existing treatments with regard to efficacy [19-21]. The methodological principles for the non-inferiority trial design are described in the International Conference on Harmonisation guideline E10 [22]. The design of a non-inferiority study requires the a priori definition of a non-inferiority margin, often called delta, which describes the threshold value of clinical relevance.

There are clinical and statistical aspects to be considered when choosing a non-inferiority margin [23]. The major clinical requirement for choosing a non-inferiority margin is that any treatment difference smaller than the non-inferiority margin should not be of clinical relevance. The major statistical requirement for choosing a non-inferiority margin is that the non-inferiority margin is small enough to exclude the effect of placebo. The focus of this paper is to empirically define the threshold value of clinical relevance for EAPP measured on a VAS that fulfils these criteria. It should be noted that the definition of the clinically relevant threshold is independent of the difference between a given treatment and placebo. However, exclusion of the placebo effect needs to be considered when applying the threshold value of clinical relevance in a clinical trial.

The data for this paper derive from two recent randomized, placebo-controlled clinical trials in EAPP. Both trials used a very similar design, which is reported elsewhere [24]. The patients recorded their EAPP on a VAS at screening, baseline, during, and at the end of treatment (week 12). At the end of the treatment, patients also rated their satisfaction with treatment using a modification of the Clinical Global Impression (CGI) scale-global improvement item [25]. This simple and well-established tool for the assessment of overall treatment effect was used as an anchor for the definition of the minimal clinically important difference (MCID) for EAPP.

## Methods

### Study design

These two international, randomized, double-blind, placebo-controlled studies investigated the efficacy and safety of two different compounds in the treatment of endometriosis.

Study 1 was conducted at 33 centers in Germany (n = 19), Italy (n = 8), and Ukraine (n = 6). Study 2 was conducted at 28 centers in the Czech Republic (n = 4), Denmark (n = 2), Spain (n = 6), Finland (n = 6), France (n = 1), The Netherlands (n = 3), and Sweden (n = 6). The study protocols were approved by local independent Ethics Committees and all participants provided written informed consent before study enrollment. The studies were conducted in accordance with the amended version of the Declaration of Helsinki and complied with Good Clinical Practice.

### Patients

**Figure 1:**
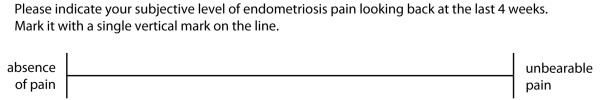
**The VAS**. Patients record the severity of their pain on a VAS score from 0 mm to 100 mm.

Women aged 18 to 45 years, between menarche and menopause and in good general health except for endometriosis, were eligible for study inclusion (Table 1). Inclusion criteria included endometriosis stage I-IV, according to revised American Society of Reproductive Medicine (r-ASRM) scoring [11], which was assessed at diagnostic laparoscopy within 12 months prior to study baseline. Patients were required at both screening and baseline to have an EAPP score of ≥ 30 mm (study 1) or ≥ 40 mm (study 2) on a VAS, where the anchor points were 0 mm (representing absence of pain) and 100 mm (indicating unbearable pain), without intervening markings (Figure 1).

**Table 1:** Patient demographics.

Total number of patients, n (%)	281 (100)
Ethnic group, n (%)	
Caucasian	278 (98.9)
Black	1 (0.4)
Asian	2 (0.7)
Age (years), mean (SD)	31.9 (6.4)
Weight (kg), mean (SD)	63.0 (10.8)
Body mass index (kg/m²), mean (SD)	22.8 (3.7)

SD, standard deviation

Exclusion criteria included pregnancy or breastfeeding, use of an intrauterine device, amenorrhea within 3 months of screening, signs or symptoms of therapy-resistant endometriosis or need for near-term surgical treatment of endometriosis, previous use of hormonal agents (e.g., gonadotropin-releasing hormone agonists ≤ 6 months before screening, progestins or danazol ≤ 3 months before screening, or oral contraceptives ≤ 1 month before screening), clinically relevant findings at gynecological examination, or an abnormal cervical cytological smear in the last 3 months.

Of 308 women with moderate-to-severe EAPP randomized in the two studies, 281 provided data on the CGI scale and change in EAPP.

### Efficacy endpoints

The primary efficacy variable in both studies was the absolute change in EAPP from baseline to the end of treatment. EAPP was evaluated at weeks 0, 4, 8, and 12 by assessment of pain score on the VAS and intake of supportive analgesic medication (ibuprofen tablets) for pelvic pain.

Secondary efficacy variables included, among others, a global assessment of efficacy by patients and investigators using the CGI scale-global improvement item [25] (Table 2), which was applied at the end of treatment.

**Table 2:** Subjects' assessments on the CGI scale-global improvement item (n, %).

CGI scale		Aggregated CGI scale	
Very much satisfied	18 (6.4)	Satisfied	108 (38.4)
Much satisfied	90 (32.0)		

Minimally satisfied	101 (35.9)	Minimally satisfied	101 (35.9)

Neither satisfied nor dissatisfied	50 (17.8)	Undecided or worse	72 (25.6)
Minimally dissatisfied	14 (5.0)		
Much dissatisfied	7 (2.5)		
Very much dissatisfied	1 (0.4)		

### Statistical methods

Following the intent-to-treat approach, all randomized patients who provided data were included in the analyses, regardless of possible protocol deviations.

EAPP was recorded by patients on a VAS before (at screening and baseline), during, and at the end of treatment. From these measurements, the individual absolute change in EAPP was derived by subtracting the baseline VAS score from the VAS score at end of treatment. At the end of treatment, patients also rated their overall satisfaction using the CGI scale-global improvement item. For one patient, a missing assessment was replaced by the corresponding physician's assessment, because these two ratings showed substantial agreement (weighted κ coefficient 0.69, n = 294; 95% confidence interval [CI] 0.64-0.75), according to the definition of Landis and Koch [26]. Missing VAS scores were not imputed. In cases where patients dropped out prematurely, the last available measurement under treatment was included in the analysis (last value carried forward method).

All variables were analyzed by descriptive statistics, either by absolute and relative frequencies for discrete data, or by the number of non-missing observations, mean, standard deviation, minimum, 25th percentile, median, 75th percentile, and maximum for metric data.

Given that several categories on the seven-point GCI scale were rarely ticked by the women (see Table 2), the scale was aggregated to a three-point scale for further analyses. The entries "very much satisfied" and "much satisfied" were merged into "satisfied" and the entries "neither satisfied nor dissatisfied" to "very much dissatisfied" were merged into "undecided or worse", whereas the category "minimally satisfied" was left unchanged. These three resulting categories were of approximately equal size and there were no relevant differences in the VAS scores for the categories merged. This one-sided approach was used because the patients reporting themselves as "neither satisfied nor dissatisfied" showed, on average, a slight improvement of their VAS scores and because this approach also conserved the direction of the changes.

A bidirectional approach was additionally added as a sensitivity analysis, with the assumption that patients rate an increase in pain in the same way as a reduction in pain. For this analysis, the CGI categories were grouped into the three categories: "much change" (including the categories "very much satisfied", "much satisfied", "much dissatisfied", and "very much dissatisfied"), "minimal change" (including the categories "minimally satisfied" and "minimally dissatisfied"), and "no change" (the remaining CGI category "neither satisfied nor dissatisfied"). VAS score changes for satisfied patients (who were assumed to have a reduction in pain) were multiplied by-1 for this analysis.

Boxplots were drawn using the 10th and 90th percentile as endpoints of the whiskers. Outlying observations were also shown, using a dot as the plot symbol. A non-parametric discriminant analysis with normal kernels and unequal bandwidths [27] was performed. A one-way analysis of variance (ANOVA) of the changes in VAS score with factor grouped CGI category was performed to estimate the mean differences and their 95% confidence intervals. All statistical analyses were performed using version 9.1 of SAS software [28], running under Windows XP Professional.

## Results

### Demography

Of the 308 women randomized in the two studies, 281 (91.2%) provided data both on the CGI scale and on their change in pelvic pain and were included in this analysis. Almost all of these women were Caucasian, with a mean age of 32 years and a mean body mass index of 23 kg/m² (Table 1).

### CGI scale-global improvement item

The global efficacy assessment (Table 2) showed that 108 (38.4%) of the women were at least "much satisfied", 101 (35.9%) were "minimally satisfied", and 22 (7.8%) were "minimally dissatisfied" or worse with their treatment. The remaining 50 (17.8%) women rated themselves as "neither satisfied nor dissatisfied".

### VAS score

The mean (± standard deviation [SD]) EAPP decreased during treatment from 58.9 ± 17.3 mm to 37.0 ± 23.6 mm on the VAS (Table 3). The mean change from baseline was -22.9 ± 22.7 mm.

**Table 3:** EAPP at baseline and end of treatment (VAS score, mm*) (n = 281).

	Mean	Standard deviation	Minimum	25th percentile	Median	75th percentile	Maximum
Baseline	59.8	17.3	20	47	60	72	98
End of treatment	37.0	23.6	0	18	34	53	100
Change from baseline	-22.9	22.7	-93	-36	-20	-8	67

*The VAS scores pain on a scale from 0 mm (absence of pain) to 100 mm (unbearable pain). A negative mean change in VAS score indicates a reduction in pain.

**Figure 2:**
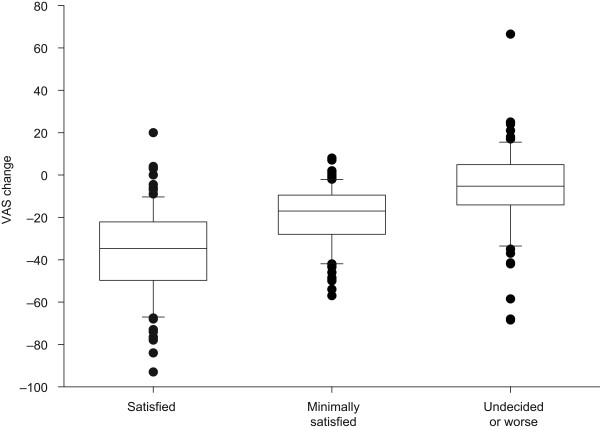
**Change in VAS score categorized by patients' assessments on the modified CGI scale-global improvement item**. Boxplots are drawn using the 10th and 90th percentiles as endpoints of the whiskers. Outlying observations are shown using a dot as the plot symbol.

The relation between the VAS scores and patients' CGI assessments is shown in Figure 2. As expected for a highly subjective measure like pain, there was some overlap between the observed changes in the VAS scores and the patients' perceptions of how their EAPP had changed. Women who were "satisfied" with their treatment according to the CGI assessment had a mean (± SD) change in VAS score of -36.9 ± 21.8 mm, women who were only "minimally satisfied" had a change of -19.5 ± 14.3 mm, and women who felt "undecided or worse" had a change of -6.5 ± 20.7 mm (Table 4). On average, women who felt "minimally satisfied" had a VAS score that was 12.8 mm lower compared with women who felt "undecided or worse". Using the bidirectional approach, mean (± SD) changes in VAS score were 33.9 ± 24.0 mm, 18.6 ± 16.3 mm, and 7.1 ± 19.5 mm for women reporting "much change", "minimal change", and "no change", respectively.

**Table 4:** Change in EAPP (VAS score, mm*), categorized by subjects' assessments on the aggregated CGI scale-global improvement item.

Subjects' assessment	n	Mean	Standard deviation of change	Minimum	25th percentile	Median	75th percentile	Maximum
Satisfied	108	-36.9	21.8	-93	-50	-35	-22	20
Minimally satisfied	101	-19.5	14.3	-57	-28	-17	-10	8
Undecided or worse	72	-6.5	20.7	-69	-14	-5	5	67

*The VAS scores pain on a scale from 0 mm (absence of pain) to 100 mm (unbearable pain). A negative mean change in VAS score indicates a reduction in pain.

The anchor-based MCID in EAPP is determined by the value of VAS score change that best separates women rating themselves as "undecided or worse" from those rating themselves as "minimally improved". This MCID of -7.8 mm was determined by non-parametric statistical discriminant analysis. The best separation between women rating themselves "improved" and those rating themselves "minimally improved" was -27.6 mm on the VAS. Using the bidirectional approach, the cutoff values determined by discriminant analysis were 8.7 mm and 28.2 mm. The difference between the means of "no change" and "minimal change" determined by ANOVA was 11.5 mm (95% CI 4.7-18.2) and the difference between the means of "no change" and "much change" was 26.8 mm (95% CI 20.0-33.6).

The distribution-based MCID is derived by halving the standard deviation of the VAS scores at baseline. According to empirical work by Norman et al. [29], this generally provides a reliable estimate for an MCID for patient-reported outcomes such as pain measurements. The standard deviation of the VAS scores at baseline was 17.3 mm (Table 3), yielding a distribution-based MCID of -8.6 mm when considering that a reduction in VAS score implies an improvement in EAPP.

## Discussion

The VAS is among the most widely used pain scales [30] and has been used in many conditions, including acute and chronic pain of various origins [31-33]. The validity and reliability of the VAS using anchor points of 0 mm (absence of pain) and 100 mm (unbearable pain) have been demonstrated for different pain indications [33] and values for the MCID have been established for different types of pain; for example, in acute abdominal pain, the MCID is reported as 13 mm (95% CI, 10-17) [34,35], while MCID values appear generally to be lower for chronic pain compared to acute pain [33].

The VAS was also commonly used in recent studies specifically designed to evaluate the pain associated with endometriosis [36-45]. However, an empirical evaluation of an MCID for this indication has been lacking. The aim of this analysis was to derive an empirically validated MCID for EAPP and compare it to the MCID reported for other pain indications. We observed an MCID of approximately 10 mm for the change in EAPP measured by VAS, irrespective of whether an anchor-based or a distribution-based approach was used and irrespective of whether a one-sided or a bidirectional approach was used.

A relatively large proportion of women in the two studies reported that they were at least somewhat satisfied with their treatment, although both studies used placebo as a control group. This observation can be explained by the relatively large placebo effect, which is in line with other well-designed studies in this indication [28,44].

Our empirical results for an MCID for EAPP measured on a VAS are comparable to the results for pain measured by VAS in other settings, e.g., pain self-assessment by patients with rheumatoid arthritis [31] or physician-assigned pain scores across different types of pain [32]. They are also in line with non-inferiority margins recently used in different chronic pain conditions, including chronic low back pain [46], osteoarthritis [47], and ankylosing spondylitis [48].

The limitations of our study were that we used only one anchor and we measured the anchor only at the end of the study. Hence the intra-subject variability of the anchor-based MCID could not be determined. Additional studies will be required to confirm that the results of our analyses are generalizable to other patient populations and other forms of endometriosis-associated pain.

The MCID of 10 mm for EAPP measured on a VAS could also be used to define a non-inferiority margin for the head-to-head comparison of two active treatments in a non-inferiority trial. In this case, the statistical requirement that the non-inferiority margin is limited by the effectiveness of the reference treatment with respect to placebo [27] has to be considered. For a meaningful result, the non-inferiority margin must be smaller than the difference between the reference active treatment and placebo.

In conclusion, the empirically validated MCID for EAPP measured on a VAS is 10 mm. This MCID could also be used to define a non-inferiority margin for a head-to-head comparison of two active treatments.

## Competing interests

All the authors except US are full-time employees of Bayer Schering Pharma AG. US is a part-time employee of Jenapharm GmbH & Co. KG. The authors have no additional financial or non-financial competing interests.

## Authors' contributions

**CG **was involved in the conception and design of this study, in the analysis and interpretation of data, and in development and review of the manuscript for intellectual content. **US **was involved in the analysis and interpretation of data, and in development and review of the manuscript for intellectual content. **TF **was involved in the interpretation of data and in review of the manuscript for intellectual content. **AC **was involved in the interpretation of data and in review of the manuscript for intellectual content. **HS **was involved in the interpretation of data and in review of the manuscript for intellectual content. **CS **was involved in the conception and design of this study, in the interpretation of data, and in development and review of the manuscript for intellectual content. All authors read and approved the final manuscript.
